# Magnetic nanocatalysts as multifunctional platforms in cancer therapy through the synthesis of anticancer drugs and facilitated Fenton reaction

**DOI:** 10.1016/j.jare.2020.12.001

**Published:** 2020-12-05

**Authors:** Suliman Khan, Majid Sharifi, Anwarul Hasan, Farnoosh Attar, Zehra Edis, Qian Bai, Hossein Derakhshankhah, Mojtaba Falahati

**Affiliations:** a Department of Cerebrovascular Diseases, The Second Affiliated Hospital of Zhengzhou University, Zhengzhou, PR China; b Department of Nanotechnology, Faculty of Advanced Sciences and Technology, Tehran Medical Sciences, Islamic Azad University, Tehran, Iran; c Department of Mechanical and Industrial Engineering, College of Engineering, Qatar University, Doha 2713, Qatar; d Biomedical Research Center, Qatar University, Doha 2713, Qatar; e Department of Food Toxicology, Research Center of Food Technology and Agricultural Products, Standard Research Institute (SRI), Karaj, Iran; f Department of Pharmaceutical Sciences, College of Pharmacy and Health Sciences, Ajman University, PO Box 346, Ajman, United Arab Emirates; g Department of Anesthesiology, The Second Affiliated Hospital of Zhengzhou University, Zhengzhou, PR China; h Pharmaceutical Sciences Research Center, Kermanshah University of Medical Sciences, Kermanshah, Iran

**Keywords:** Magnetic nanocatalysts, Heterocyclic compounds, Anticancer drugs, Fenton reaction, Cancer therapy

## Abstract

**Background:**

Heterocyclic compounds have always been used as a core portion in the development of anticancer drugs. However, there is a pressing need for developing inexpensive and simple alternatives to high-cost and complex chemical agents-based catalysts for large-scale production of heterocyclic compounds. Also, development of some smart platforms for cancer treatment based on nanoparticles (NPs) which facilitate Fenton reaction have been widely explored by different scientists. Magnetic NPs not only can serve as catalysts in the synthesis of heterocyclic compounds with potential anticancer properties, but also are widely used as smart agents in targeting cancer cells and inducing Fenton reactions.

**Aim of Review:**

Therefore, in this review we aim to present an updated summary of the reports related to the main clinical or basic application and research progress of magnetic NPs in cancer as well as their application in the synthesis of heterocyclic compounds as potential anticancer drugs. Afterwards, specific tumor microenvironment (TME)-responsive magnetic nanocatalysts for cancer treatment through triggering Fenton-like reactions were surveyed. Finally, some ignored factors in the design of magnetic nanocatalysts- triggered Fenton-like reaction, challenges and future perspective of magnetic nanocatalysts-assisted synthesis of heterocyclic compounds and selective cancer therapy were discussed.

*Key Scientific Concepts of Review:*

This review may pave the way for well-organized translation of magnetic nanocatalysts in cancer therapy from the bench to the bedside.

## Graphical abstract

**Figure:**
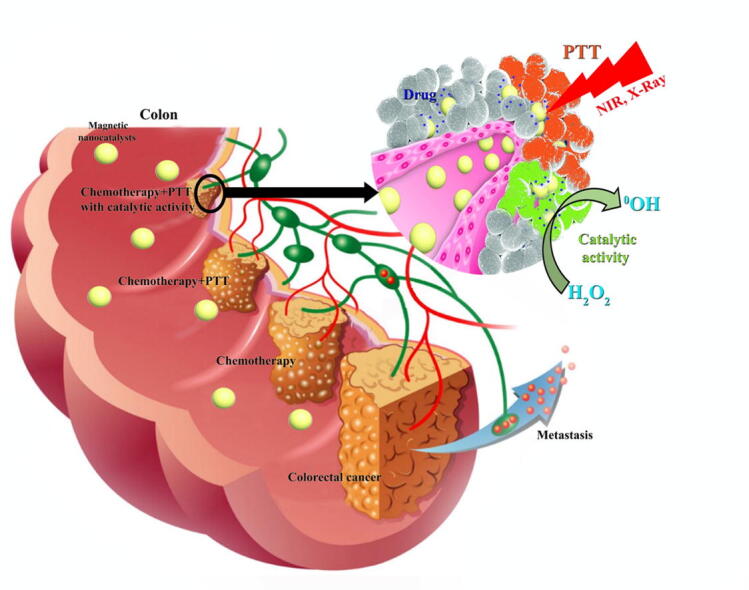

## Introduction

Catalysts are widely used in the production of chemicals [1], [2] and pharmaceutical ingredients [3], [4]. A large number of products require the application of catalysts; where, it has been estimated that a large portion of manufactured products of industrialized countries' GDP is dependent on catalysts [5]. Today, researchers have a big effort to incorporate nanomaterials into catalysts as a successful and promising strategy in the development of nano-based platforms. Because of their great significance of nanocatalysts in different areas of nanotechnology such as nanochemistry, nanopharmaceuticals, and nanomedicine, the number of articles and books published about their application is continuously increasing. Nanomaterials show distinctive catalytic characteristics and are widely used in the preparation of nanocatalysts [6], [7]. Indeed, many of the properties of materials such as electrical, optical, and magnetic behaviors change at nanoscale dimension [8], [9]. These novel characteristics are derived from the following three properties: small size less than 10 nm, potential high surface (S)/volume (V) ratio, and an increase in the number of surface atoms. In fact, these three factors are the most important reasons for the emergence of catalytic properties in nanomaterials. Basically, when particles become small (nanoscale), because of their high curvature, they have many atoms on their surface establishing weak bonds with the lattice atoms [10]. Therefore, these particles have high surface energy and are highly active, so-called surface atoms are physically unstable and chemically active and are prone to many chemical reactions [11], [12]. Since the electronic structure of the NPs depends on the dimension of the particle, their capability to interact with other compounds also depends on their dimension [13], [14]. For example, one group of materials that behave differently in bulk and nanoscale forms is magnetic particles. Magnetic particles have a limited catalytic activity and are considered as inactive metals in the bulk state, but at the nanoscale, they exhibit profound catalytic activity and are commonly used as intermediate metals in the development of nanocatalysts [15], [16].

The catalyst design at the NP scale is heavily based on the principles of size [17] and mass transfer [18]. Recent advances have shown that the amelioration of the catalytic activities of catalysts can be achieved through nanoscale structural modification [19], [20]. Indeed, due to the high cost and scarcity of the most catalytically active metal group, there has been a great interest in the use of these materials with low concentration and optimized catalytic activity. At the nanoscale, the properties of materials are dictated by the arrangement of atoms [21]. Table 1 summarizes the factors affecting the catalytic properties of nanomaterials.

**Table 1:** Factors influencing the catalytic properties of nanomaterials.

**Factors**	**Explanation**	**Ref.**
NP size	In most cases, the catalytic activity of NPs is inversely related to the size of NPs	[22]
NP shape	As the spatial distribution of the NPs increases, the number of surface atoms becomes more available which result in the enhancement of the catalytic activity	[22]
NP distribution	NPs with many edges and corners, such as tetrahedral, octahedral, and cubic, as the ratio of surface to volume increases more than other NPs, these nanomaterials show more catalytic activities.	[23]
NP supports	The solid substrate can be used for immobilization of NPs to prevent the NPs from accumulating and their so-called agglomeration and increase their catalytic activity	[24]
Reaction conditions	When NPs react under microwave conditions, the catalytic activity and selectivity over normal conditions (reflux) are greatly improved.	[25]

## Different kinds of magnetic nanocatalysts

Despite the variation in the classification of magnetic nanocatalysts due to different physicochemical properties of NPs, the method of fabrication, surface modification, crystal structure, and composition of magnetic nanocatalysts derived from iron (Fe), gold (Au), palladium (Pd), and platinum (Pt) can be organized based on their oxidase-, peroxidase-, superoxide dismutase-, and catalase-like activities (Table 2).

**Table 2:** Summary of magnetic nanocatalysts based on their intrinsic activity.

**NPs**	**Oxidase**	**Peroxidase**	**Superoxide dismutase**	**Catalase**
Fe	FeNPs	Fe_3_O_4_, Fe_2_O_3_	FePO_4_	Fe_3_O_4_, Fe_2_O_3_
Au	AuNPs, Au@Pt	AuNPs, Au@Pt	AuNPs,	AuNPs,
Mn	MnO_2_, Mn_3_O_4_	MnO_2_, Mn_3_O_4_, MnFe_2_O_4_	MnO_2_, Mn_3_O_4_	MnO_2_, Mn_3_O_4_
Ni	NiPd	NiPd		NiPd
Ag	AgNPs	AgNPs	AgNPs	AgNPs
Pt	PtNP, PtCo	PtNP, PtNPs/GO	PtNP	PtNP
Pd	PdNP	PdNP	PdNP	PdNP
Co	CoFe_2_O_4_	Co_3_O_4_, CoFe_2_O_4_		Co_3_O_4_

However, the most commonly used magnetic nanomaterial is iron oxide (IO), whose most important catalytic activities in medical activities are peroxidase-, superoxide dismutase-, and catalase-like activities (Table 3). Peroxidase is the most well-known enzymatic activity mimicked by IO nanocatalysis. For peroxidase activity, magnetic nanocatalysts similar to the Horseradish peroxidase (HRP) require an optimum temperature of 37 to 40 °C with a pH of 3 to 6.5. Moreover, in order to increase the peroxidase activity of NPs, the presence of the optimum concentration of hydrogen peroxide (H_2_O_2_) is important.

**Table 3:** Metallic nanocatalysts and thier parameters in medical platforms.

**Material**	**Size (nm)**	**Shape**	**Activity**	**Ref.**
Fe				
Fe_3_O_4_	13	Polyhedral	Peroxidase	[26]
Fe_3_O_4_	–	Spherical	Catalase	[27]
GO-Fe_3_O_4_	6–8	NPs	Peroxidase	[28]
Fe_3_O_4_@Pt	5–10	Spherical	Peroxidase	[29]
Fe_3_O_4_@Cu@Cu_2_O	20–40	Spherical	Peroxidase	[30]
GO-Fe_2_O_3_	–	NPs	Catalase	[31]
PB-Fe_2_O_3_	30	NPs	Peroxidase	[32]
Pd@γ-Fe_2_O_3_	14–25	Polyhedral	Peroxidase	[33]
Au				
Au_2_O_3_	50	NPs	Peroxidase	[34]
AuNPs	15–34	Spherical	Multi	[35]
Au@PVP NPs	1–3	NPs	Oxidase	[36]
Pt				
PtNPs	3.8	NPs	Oxidase	[37]
PtNPs	1.2	Nanoplate	Oxidase	[38]
PtNPs	40	Multi-octahedra	Oxidase	[39]
Pd				
AgPd-GO	7–10	NPs	Peroxidase	[40]
Pd@MIL-101	1.4–1.8	NPs	Hydrolysis	[41]
PdNPs	1.5	NPs	Peroxidase	[42]
Rhodium (Rh)				
Rh-SiO_2_ NPs	5–15	Tetrahedral	Oxidase	[43]
RhNPs	1–2	NPs	Peroxidase	[44]
Rudium (Ru)				
RuNPs	1.1	NPs	Peroxidase	[45]

## Function of magnetic nanocatalysts

Magnetic NPs as catalysts have different capabilities in medical fields such as diagnosis [46], imaging [47], drug delivery [48], drug discovery [49], and cancer therapy [50] due to the inherent enzymatic activity. Since catalytic activities in chemistry are highly surface dependent, the use of metallic NPs is of particular importance due to their highly active surface [51]. Although the reaction kinetics of magnetic nanocatalyst-based catalysts are slightly lower than those of native enzymes [52], resistance to environmental changes such as heat and acidity, easy separation, excellent reusability, and cost effective have made them potential candidates in different applications [15], [53]. On the other hand, the occurrence of dual behavior of magnetic nanocatalysts or the bridge between homogeneous and heterogeneous catalysis by nanocatalysts [54], as well as the provision of a platform to induce a certain reaction like photooxidation have encouraged scientietis to use magnetic nanocatalysts [55]. Hence, a large number of magnetic nanocatalysts have been produced in the industry to catalysis the synthesis of different chemicals or drugs. Magnetic nanocatalysts in the form of homogeneous and heterogeneous catalysts have been used in biomedical activities [56], [57], among which IONPs have the highest application due to their very low toxicity [58]. Furthermore, magnetic nanocatalysts are highly considered in therapeutic platforms, especially in cancer therapy, anti-inflammatory activities, antibacterial, tissue engineering, and immunotherapy. The integration of the homogeneous and heterogeneous catalysts in biomedical fields enabled by medical nanocatalysts through light and magnetic waves has provided a potential system for development of therapeutic platforms.

## Main clinical or basic application and research progress of magnetic NPs in cancer

Before magnetic NPs were considered as catalysts, they were used in the fields of chemotherapy [59], imaging [60], gene therapy [61], photothermal therapy (PTT) [62], magnetic hyperthermia [63], radiation therapy [64], and photodynamic therapy (PDT) [65]. The drug delivery by magnetic NPs has become a popular way to transfer drugs to the tumor site due to controllable physicochemical properties. For example, the loading and delivery of paclitaxel [66], doxorubicin [62], [63], 5-fluorouracil [67], and even small molecules or proteins such as lactoferrin [68] or Bcl-2 shRNA [69] to tumors has been successfully conducted. Furthermore, evaluation of vital organs around the target tissue has shown that magnetic NPs have triggered a minor effect on the pathological changes and systemic toxicity [62], [63], [70], [71], [72]. It has been shown that the use of magnetic NPs enhances the therapeutic activity against tumors by inducing more damage to DNA, while NPs activated by radiotherapy did not show such toxicity on the cancer cells [73]. Moreover, magnetic NPs significantly sensitize the tumor cells to radiation therapy [74] by inducing a hypoxic condition for the generation of active oxygen.

Furthermore, it was found that the use of magnetic NPs, in addition to improving cancer immunotherapy through facilitating the antibody penetration into the tumor, increases the quality of imaging of target tissue by MRI [75]. Furthermore, different types of magnetic NPs have shown anti-cancer activities. In this regard, Zanganeh et al. [76] showed that ferromoxytol-functionalized IONPs can trigger potential anticancer activity against lung, liver and breast cancers.

Thermal therapy enabled by magnetic NPs for treatment of cancer has received much attention due to their high efficiency and very low toxicity in critical tissues [50]. The most important methods of tumor ablation therapies through increase in free radical species enabled by magnetic NPs include magnetic hyperthermia, PTT [62], [63], [77], and PDT [78]. Despite the methods mentioned in the treatment of cancer with magnetic NPs, today the combination of these methods with the catalytic activity of magnetic NPs is done in order to make the primary treatment easier or more effective. For example, Nie et al. [79] showed that CuS-Fe@polymer nanocatalysts highly improved chemodynamic therapy through PTT and peroxidase-like activity in tumor site. Similarly, Zhao et al. [80] by developing ROS-activatable liposomes@oxaliplatin@Fe_3_O_4_ nanocatalysts designed a platform for synergistic photo/chemodynamic therapies.

## Magnetic nanocatalysts in the synthesis of anticancer drugs

NPs can be used as promising platforms to accelerate the process of converting organic compounds to one another [81]. These include organic compounds widely used in chemical and pharmaceutical industries [82]. As heterocyclic compounds with anticancer properties widely used in the pharmaceutical industry, an easy, efficient and environmentally friendly method for synthesizing these organic compounds has been introduced by using nanocatalysts [83], [84].

### Nanocatalysts in fabrication of heterocyclic compounds as novel anticancer drugs

Heterocyclic compounds are known to act as promising anticancer drugs [85]; where, they have been reported as the key structural components of several anticancer agents present on the market, recently. Indeed, of the potential anticancer drugs approved by the FDA between 2010 and 2019, about 40% had heterocyclic compounds within their formulations. The importance of this plan is in the feasible and inexpensive synthesis of these compounds using nanocatalysts and the investigation of their structural characteristics and performance through computational studies. Expensive homogeneous catalysts, multistage and complex manufacturing processes are commonly used in drug production processes; however, the application of nanocatalyst in the development of anticancer drugs is highly efficient and has the ability to be reused in the drug manufacturing process [86]. The nanocatalysts decrease production costs, increase biocompatibility via the use of non-toxic metals and non-volatile solvents, and limit the production of by-products [87]. Thereby, nanocatalyst can not only be extracted from the inexpensive sources, but they also are resistant to air and moisture and exhibit potential catalytic performance in chemical reactions [88]. Also, heterocyclic compounds in the presence of nanocatalysts can be synthesized via different routes [86], [89].

**Fig. 1:**
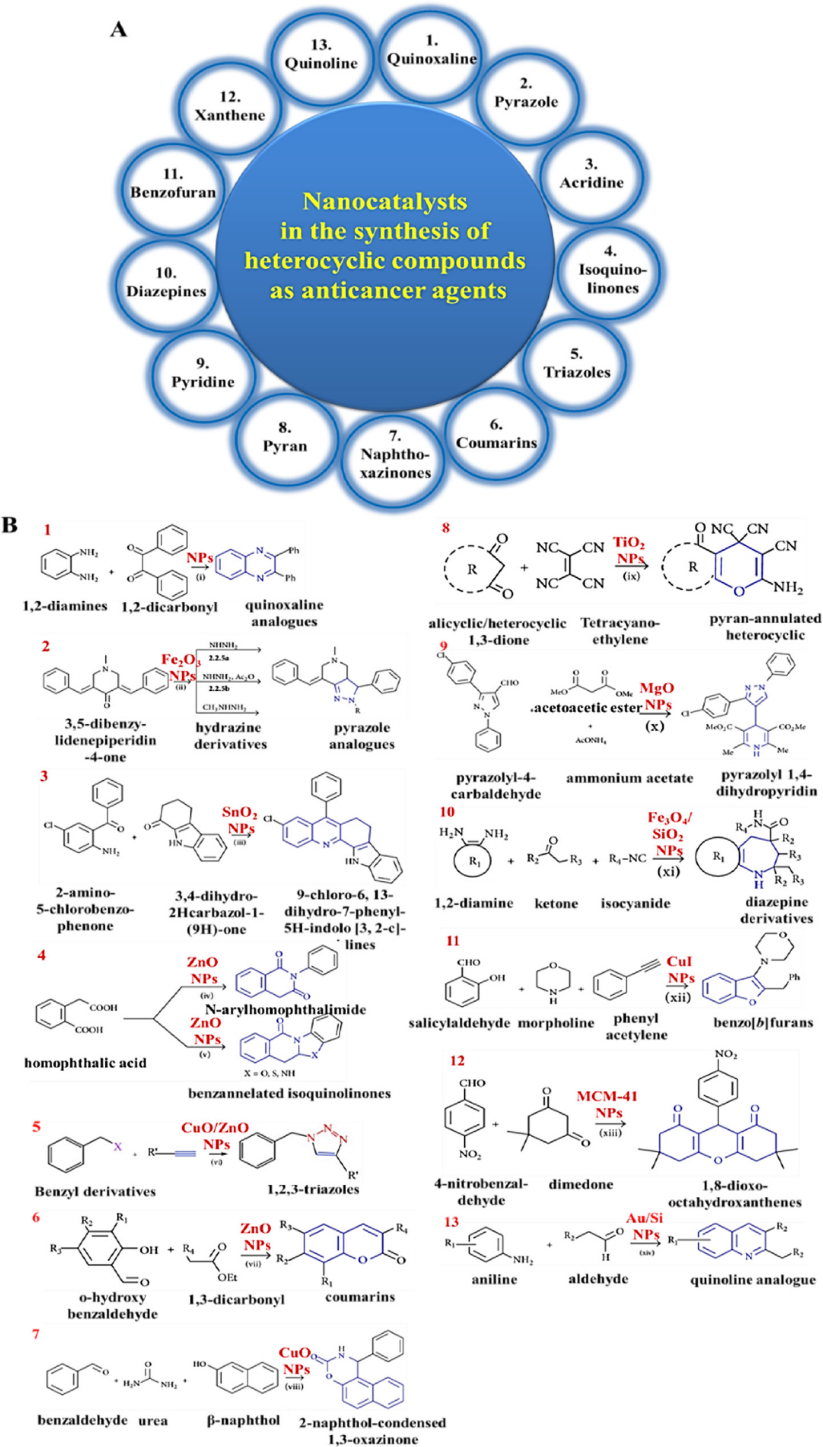
(A (Schematic illustration of using nanocatalysts in the synthesis of different heterocyclic compounds. (B(The reactions of synthesis of heterocyclic compounds. Adapted with permission from Ref. [90].

Quinoxaline, Pyrazole, Acridine, Isoquinolinone, Triazoles, Coumarins, Naphthoxazinones, Pyran, Pyridine, Diazepines, Benzofuran, Xanthene, and Quinoline are the most common heterocyclic compounds used as anticancer agents (Fig. 1A) [90]**.**

These compounds can be synthesized in a variety of mechanisms by using different nanocatalysts (Fig. 1B). One of the performances of synthesized pharmaceutical compounds can be highlighted as following: estrogen receptors (ER) and aromatase enzymes are two important factors in cancer therapy, more precisely in breast cancer; hormone therapy can inhibit estrogen production by blocking aromatase or using ligands to block estrogen receptors and stopping estrogen activity and cell growth [85]. The other mechanisms of anticancer properties of heterocyclic compounds are tabulated in Table 4.

**Table 4:** Mechanism of different heterocyclic compounds in cancer therapy.

**Heterocyclic compounds**	**Drugs approved by FDA**	**Type of cancer**	**Mechanism of anticancer**	**Ref.**
**Nitrogen-based heterocycles**	Vincristine and vinblastine	Acute lymphoblastic leukemia, Hodgkin’s and non-Hodgkin’s lymphoma, and testicular cancer	Inhibition of cell signaling, cell cycle arrest, inhibition of tumor vascularization and DNA repair, induction of oxidative stress, tubulin depolymerization	[91]
Oxygen-based heterocycles	Cabazitaxel and eribulin	Prostate and metastatic breast cancer	Depolymerisation of microtubule, inhibition of mitosis	[92]
Sulfur-based heterocycles	Dabrafenib	Melanoma, lung cancer	Inhibition of tyrosine kinase	[93]

The molecules synthesized in the presence of nanocatalysts show similar size, shape, polarity, and cytotoxic effects against cancer cells as compared to classical compounds. Potentially, the interaction of these compounds with the estrogen receptor have been further investigated through molecular docking calculations and density-functional theory (DFT) studies and yielded favorable results. Therefore, in the presence of the nanocatalyst, the anticancer drug can be prepared in a simple and inexpensive way [85]. Also, the synthesis of heterocyclic compounds by using nanocatalysts shows several advantages such as short reaction time, high throughput, and solvent-free medium [84]. A range of nanocatalysts such as magnesium oxide [94], SBA‐15 [86], [95], cobalt [96], palladium [97], [98], copper [99], graphene oxide [100], [101], and carbon nanotubes [102] have been also used as promising and efficient agents for synthesis of heterocyclic compounds with potential anticancer effects [103], [104], [105].

### Magnetic nanocatalysts: Homogeneous or heterogeneous catalysis in drug synthesis

Also, the main strategy of catalysis science and technology is to increase the catalytic function and selectivity as well as recovery of the nanocatalysts. Indeed, the recovery and reutilization of nanocatalysts seem to be crucial parameters owing to strict biological and economical demand for sustainability [106], [107], [108]. Homogeneous catalysts show several positive points that they are well-structured on a molecular basis and easily dissolvable in the reaction milieu [54]. Therefore, these catalysts are extremely approachable to the substrates and normally demonstrate profound catalytic function, even under harsh environments. However, their separation from the reaction medium to circumvent the interactions with product needs exorbitant and repetitive extraction processes. Moreover, the inorganic nanocatalysts are usually composed of an expensive noble metal. Hence, in spite of their innate superior points, homogeneous catalysts are rarely applied in pharmacologically or medically applicable systems. On the other hand, there are frequently catalytically functional sets with several activities and specificities in the material of a heterogeneous catalyst, which are not suitable to be addressed in the molecular approach. Therefore, purification and reutilization of homogeneous catalysts is a principal concern in the sustainable and wide-ranging fabrication of organic compounds [54]. The catalyst can be purified through dissolving products and nanocatalysts in separate non-miscible solutions, which result in the purification of nanocatalysts by uncomplicated phase separation. However, the solubility of reactants in the reaction milieu and the mass transfer should be considered in the purification process [54]. Also, the activity of the purified catalyst may be partially inhibited by increasing their tendency to agglomeration during purification processes [109]. Nanocatalysts can be easily solubilized in a liquid solution to form a stable nanosuspension; however, during conventional purification process, they can be aggregated into particles with diameters of more than 100 nm. Therefore, to overcome this circumstance, ultracentrifugation is normally applied as the only way to purify the nanocatalyst. However, this approach needs high-priced and high technology facilities to achieve the potential results. Thus, magnetic nanocatalysts, which can be readily purified from the reaction medium by magnetic utilization, can be introduced as promising nanocatalysts in the pharmacological industry.

Therefore, catalytic capabilities of magnetic NPs consisting of Fe_2_O_3_ and an organic component are further investigated to accelerate the introduced production process. Also, the magnetic nanocatalysts provide a higher activity than their bulk counterparts due to its high S/V ratio, which greatly increases the efficiency of chemical synthesis process. In addition, having magnetic properties enables them to be separated from the manufactured product upon completion of the reaction. Therefore, the final product purification process will be performed with greater ease and speed [110]. For example, magnetic nickel-Fe_2_O_4_ nanocatalysts were employed as effective and reusable nanocatalyst for fabrication of acetylferrocene chalcones as potential anticancer candidates against colon cancer (HCT116), breast cancer (MCF7), and liver cancer (HEPG2) [111]. Therefore, it may be concluded that different species of magnetic NPs can be utilized as recoverable nanocatalysts in the synthesis of heterocyclic drugs both in solvent-free and aqueous media [112], [113], [114], [115].

## Magnetic nanocatalysts-facilitated Fenton reaction for cancer therapy

As current cancer therapeutic strategies may stimulate some adverse effects against surrounding normal tissues and/or trigger unwanted tumor metastasis, to improve the anti-tumor therapeutic efficacy, targeting tumor microenvironment (TME) has been used for cancer therapy. Indeed, in TME, the metabolism, intermediates and pH are considerably different from those in non-cancer cells. Therefore, TME-responsive nanocatalysts for cancer therapy have been recently evaluated *in vitro* and *in vivo*.

In fact, it may be suggested that if the intrinsic properties of the TME in the presence of nanocatalysts could activate the Fenton reaction, the potential cancer therapy can be achieved with minimized side effects against off-targets.

Metal NPs like IONPs have been shown to provide multiple enzyme-like functions in a pH-dependent fashion. For example, these NPs could mimic a catalase-like activity to convert H_2_O_2_ into safe H_2_O and O_2_ at pH 7.5. More fascinating, they could mimic a peroxidase-like activity to transform H_2_O_2_ into highly hydroxyl radicals (OH^•^) at acidic pH. Therefore, IONPs are believed to serve as promising nanozymes in augmentation of the anti-tumor therapeutic efficacy of OH^•^, because such a site-selective production of the highly toxic radicals could trigger the induction of apoptosis in acidic cancer cells and leave the normal cells undamaged. Indeed, magnetic nanocatalysts could potentially stimulate the cancer cells-specified Fenton reaction to produce plentiful toxic OH^•^ in the acidic TME.

According to Fenton reaction that was first introduced in 1894 by H. J. H. Fenton, H_2_O_2_ can be converted to toxic OH^•^ in the presence of ferrous (Fe^2+^/ Fe^2+^) ions [116]. This catalytic reaction has been widely investigated in the various areas for instance elimination of organic pollutants from water through decomposition of contaminants into harmless materials like water, inorganic salts and so on [116], [117].

**Fig. 2:**
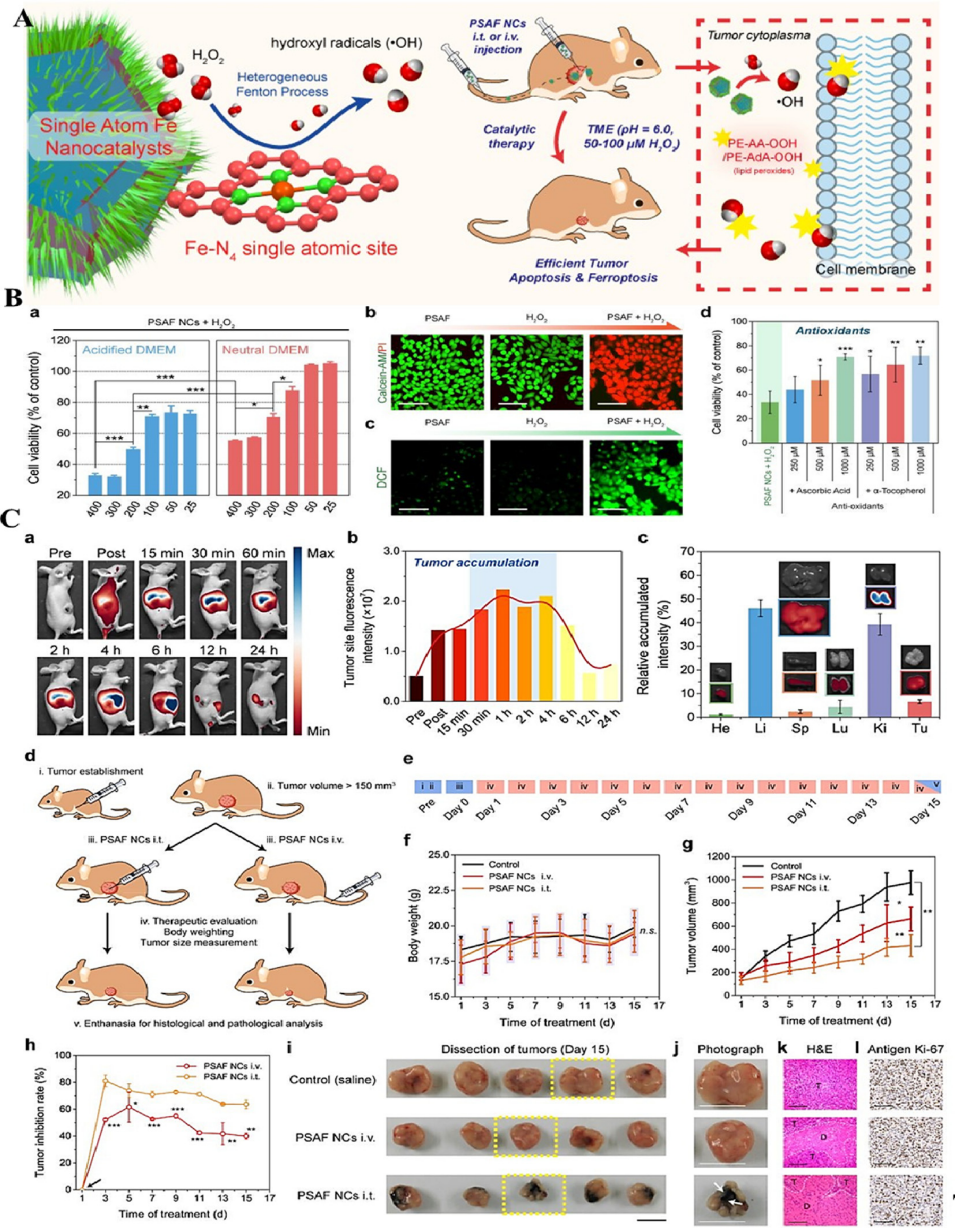
(A(Schematic representation of PEGylated single-atom Fe (PSAF) NCs in TTE. (B(Cell viability assays. Viability assays of tumor cell (4 T1) in the presence of PSAF NCs as well as H_2_O_2_ at 2 different pHs (a), Calcein-AM/PI cell staining and (b) and DCFH-DA cell- staining (c), viability percentage of the cells pre-incubated with antioxidants (d). (C) *In vivo* biodistribution and anti-tumor activity of PSAF NCs. *In vivo* biodistribution of NCs (a, b, c), *in vivo* treatment timescale of PSAF NCs (d, e), body weight (f) and tumor volume (g). tumor inhibition rates (h), Digital photos (i-j), and histological and staining microscopic images (k, l) [124]. Reprinted with permission from Ref. [124].

Fenton reaction in the cancer cells termed ferroptosis, which depends on Fe and reactive oxygen species (ROS) [118]. Indeed, in cancer cells the H_2_O_2_ molecule acts as a reactant to trigger the ferroptosis and the Fe^2+^ is the catalytic agent. It is well-documented that acidic condition is desirable for Fenton reaction; therefore, the acidic pH of TME can be considered as an effective characteristic to trigger the Fenton reaction [119]. Fig. 2(A), schematically illustrates the Fenton reactions in cancer cells.

A lot of researchers have focused on the design and development of nanostructures, which are able to induce ferroptosis reactions in different cancers [80], [120]. Based on the literature review, Fe-based nanostructures are appropriate candidates in specific accumulation at tumor region through passive as well as active targeting mechanisms. Indeed, these nano-based platforms are degraded in endocytic organelles of cancer cells in the form of Fe^2+^ or Fe^3+^ to initiate the ferroptosis process [121], [122]. In general, this catalytic process leads to imbalance between production and destruction of ROS that in turn can stimulate the severe oxidative stress-induced apoptosis. In fact, induction of intratumoral Fenton reaction can result in the disproportionation of H_2_O_2_. Therefore, development of the efficient ferroptosis-based nanocatalysts that possess transformation capacity of endogenous H_2_O_2_ to OH^•^ is an outstanding strategy for cancer therapy [123].

Besides, regarding to the stronger oxidation capacity of OH^•^ compared to singlet oxygen, the generation of these species are highly demanded for Fenton reaction. On the other hand, shelf life of OH^•^ is very short (9–10 s), which can trigger only a few oxidative reactions such as DNA damage, lipid oxidation, and protein oxidation, whereas their diffusion into remote sites is difficult [125]. Considering the aforementioned explanations, Fenton-based nanocatalysts which serve as tumor-selective nano-based platforms would be preferred for cancer therapy.

In general, it has been reported that free radicals could trigger apoptosis induction in malignant cancer cells and subsequent magnificent tumor suppression (Fig. 2A-C) [124].

**Fig. 3:**
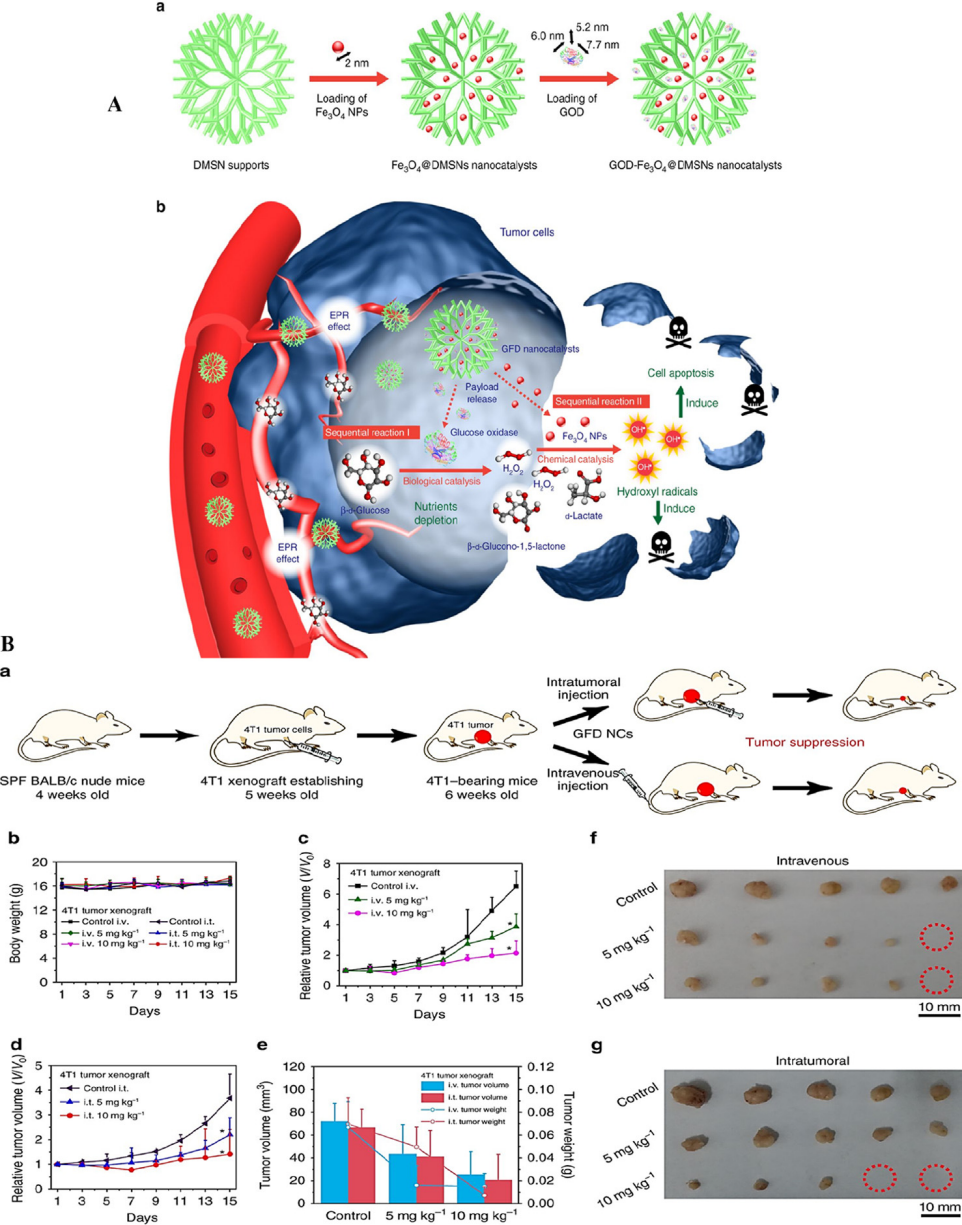
(A) Schematic representation. Fabrication (a) and catalytic-therapeutic activities of GFD NCs (b). (B) anticancer activity of fabricated GFD NCs. Schematic presentation of tumor xenograft induction and NCs administration routes, and therapeutic results (a). The body weights after treating with NCs intravenously (i.v.) and intratumorally (i.t.) (b), The relative tumor volumes mice treated with NCs via i.v. (c) and i.p. modes (d). The tumor volumes and weights of tumors (e), image of tumors after i.v. (f) and i.p. treatments (g) [126]. Reprinted with permission from Ref. [126].

Nevertheless, the intracellular level of H_2_O_2_ in cancer cells is not high enough for nanocatalysts to produce a large amount of OH^•^ to ameliorate Fenton chemical reaction for nanocatalytic tumor therapy. Therefore, a satisfactory approach to increase the intratumoral level of H_2_O_2_ was developed by Huo et al. [126]. Indeed, they reported that glucose oxidase (GOD) should be combined with nanocatalyst to increase the level of H_2_O_2_. Afterwards, IONPs integrated into the dendritic mesoporous silica NPs (DMSNs) to increase their dispersion and the ability to convert H_2_O_2_ to OH^•^ with high efficacy, which could further stimulate the anticancer activity (Fig. 3A, B).

**Fig. 4:**
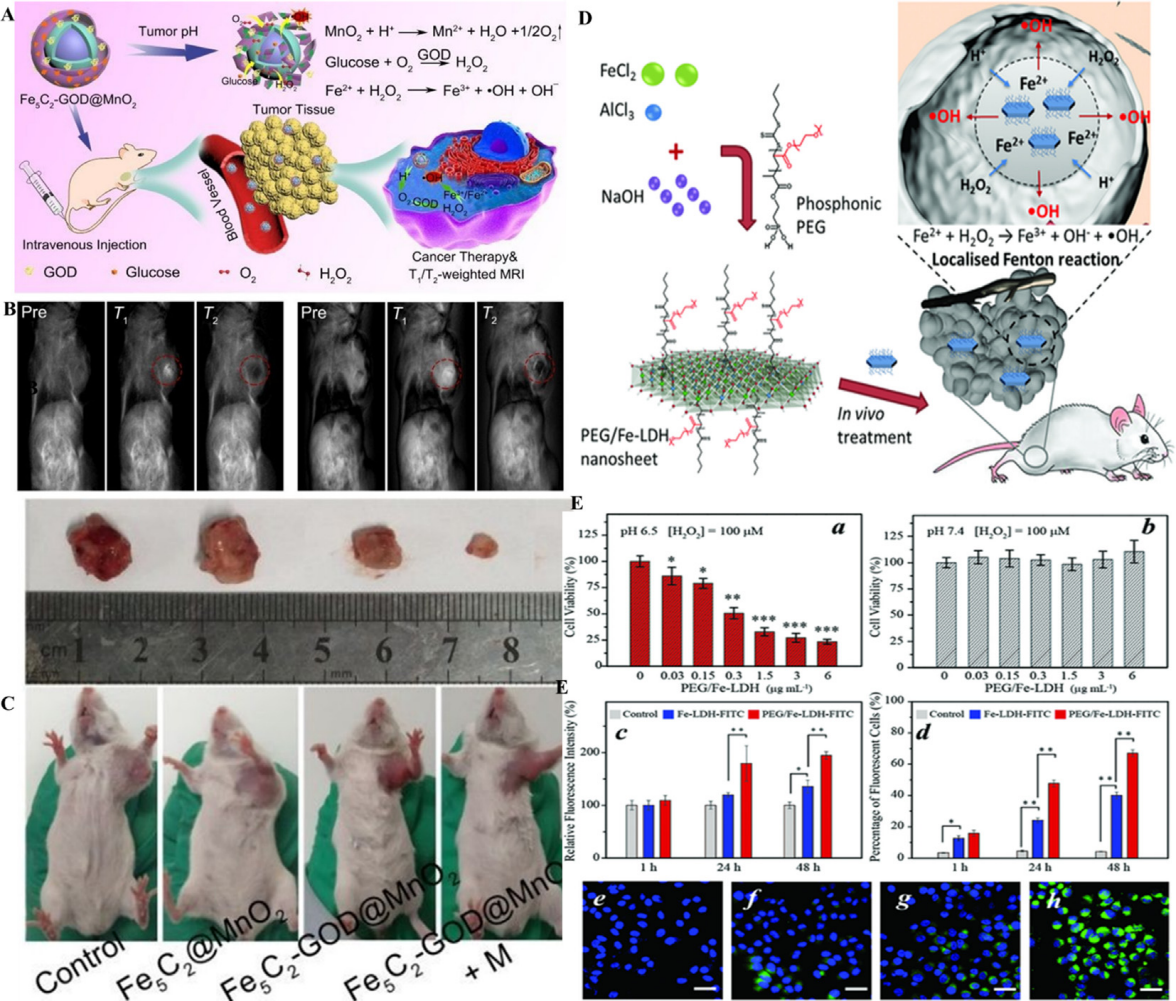
(A) Design and preparation of Fe_5_C_2_-GOD@MnO_2_ nanocatalysts. (B) *In vivo* T1/T2 MR images. Tumor bearing mice with i.v. injected Fe_5_C_2_-GOD@MnO_2_ either alone (a) or presence (b) of MF. (C) Digital images of the dissected tumors after 14 days treatment [127]. (D) Schematic illustration of TME of 2D PEG/Fe‐LDH nanosheets. (E) In vitro assays. Cell viability assays at different pH of 6.5 (a) and 7.4 (b), relative fluorescence intensity (c), and percentage of fluorescence intensity (d) based on cellular internalization, images of DCFH‐DA and DAPI staining incubated with PEG/Fe‐LDH, (e), H_2_O_2_ (f), PEG/Fe‐LDHs and H_2_O_2_ at pH 7.4 (g) and pH 6.5 (h) [76]. Reprinted with permission from Refs.[76], [127].

The strategy was proposed by Huo et al. [126] can be hampered by unwanted distribution of nanocatalyst in normal tissues and inactivation of GOD or leaking before reaching the carrier to the targeted site *in vivo*. Therefore, a gatekeeper cover can be designed as a shield on the surface of the carrier in a pH-sensitive manner to release GOD at the tumor site. Also, GOD needs oxygen to catalyze the conversion of glucose to H_2_O_2_ and carrier should provide a large amount of oxygen. The study done by Feng et al. [127], which reported the fabrication of a potential nanocatalyst equipped with a gate keeper and a source of oxygen, was performed to overcome these limitations. They constructed smart, magnetic targeted, and TME-responsive nanocatalysts that can cause oxidative stress-mediated apoptosis in tumor cells. Indeed, application of magnetic field (MF) can result in the targeted accumulation of magnetic nanocatalyst [127]. The magnetic core of IO carbide (Fe_5_C_2_)-GOD and the manganese dioxide (MnO_2_) nanoshell as a smart “gatekeeper” mask GOD from unwanted leakage until entering cancer cells. The Fe_5_C_2_-GOD@MnO_2_ nanocatalysts did not show any activity in off-targets, whereas in cancer cells, TME triggered conversion of MnO_2_ shell into Mn^2+^ and O_2_ along with leaking GOD (Fig. 4A). Mn^2+^ could be used as a magnetic resonance imaging (MRI) contrast material and O_2_ in the presence of GOD could be converted into H_2_O_2_ which may speed up the following Fenton reaction catalyzed by the Fe_5_C_2_ core (Fig. 4B, C).

Another approach to increase the amount of OH^•^ is to use 2D nanocatalyst. In this case, 2D hydroxide nanosheets can be integrated with magnetic nanocomposite to yield abundant OH^•^ in the acidic state of TME. For example, Cao et al. [128] developed a conjugated Fe^2+^‐containing double layers of hydroxide (LDH) nanosheet through a simple and useful strategy with profound catalytic function to disproportionate H_2_O_2_ in cancer cells, and subsequently produce a high amount of OH^•^ at mildly acidic milieu (Fig. 4D). Also, nanocomposites can be functionalized with PEG to achieve more biocompatibility and increase the level of internalization into the cells (Fig. 4E) [128].

## Ignored factors in design of Fenton-based nanocatalysts

In agreement with reported studies, Fenton nanocatalysts could effectively trigger the production of highly toxic OH^•^ to suppress the tumor growth in acidic TME. This phenomenon does not occur in normal tissue under neutral conditions, representing the low adverse effect of these nanoplatforms against off-targets. Nevertheless, it should be considered that cancer therapy based on Fenton nanocatalysts is a newborn approach and still remains some crucial issues to introduce this strategy to the clinical translations [129]. In the following, we will represent several important ignored factors in relationship with design of Fenton nanocatalysts for cancer therapy.

Intrinsic instability over a long period of time is one of the major problems, which can be correlated with the high tendency of nanocatalysts to form aggregated species with aim of their reduced surface energy. Another critical issue for these nanocatalysts is ease of their oxidation in air, which limits their storage for a long period of time. Reduction in interfacial area is consequence of aggregation of these magnetic nanostructures that subsequently lead to the loss of their magnetism as well as dispersibility properties [130].

To date, various strategies are applied to synthesize the magnetic nanocatalysts with high catalytic activity to provide more catalytic-active sites through the reduction of particle size or the fabrication of amorphous nanostructures [123], [131]. In spite of the presence of various routes for fabrication of nanocatalysts, their upscale production is extremely controversial.

Since, the catalytic properties of different nanocatalysts do not follow a similar mechanism and investigation of these mechanisms is not fully understood in the reported studies, the optimization of their catalytic efficacy is another critical issue that is overlooked in the most of performed studies. Therefore, catalytic mechanisms of nanocatalysts for cancer therapy should be carefully assessed in the future research.

The next issue to consider in the following research is feasibility of characterization of catalytic reaction *in vivo*. Due to the complications of and complex intracellular circumstances as well as lack of acceptable procedures at current time to characterize the catalytic mechanism *in vivo*, constructing a standard protocol for carefully characterization of catalytic reactions at *in vivo* microenvironment is more essential to conduct the future investigations in this field.

The surface engineering of nanocatalysts is a key prerequisite for their potential tumor accumulation and subsequently for achievement to excellent therapeutic efficacy. It is well documented that nanocatalysts could circumvent the reticuloendothelial system uptake via a suitable surface modification, leading the long-term blood circulation of designed structure [122]. Based on the performed literature review, the surface-engineering methodologies for design of targeted Fenton nanocatalysts are much less have been investigated [122], [132]. Therefore, efficient strategies should be established for surface modifications of Fenton nanocatalysts to improve the nanocatalytic-therapeutic efficacy in the following studies.

The next critical parameter should be noted for design of effective Fenton nanocatalysts is their biocompatibility and biosafety, guaranteeing their clinical translations in future. Although, several reports have demonstrated good biocompatibility of some Fenton nanocatalysts like Fe-based nanostructures and also their composites [133], [134]. their cytotoxicity and adverse biological effects in long periods have not been well-explored. Moreover, most Fenton nanocatalysts with high stability possess low biodegradation rates. Hence, it is expecting to optimize the biodegradation rate of these nanocatalysts and also their elimination from blood circulation for their clinical translations.

Generally, further investigations must be conducted to resolve the aforementioned challenges of Fenton nanocatalysts for cancer therapy in clinical trials.

## Challenges and future prospects

Regarding the challenges of nanocatalyst in cancer therapy it should be noted that the intracellular level of H_2_O_2_ in cancer cells should be increased to produce a large amount of OH^•^ to stimulate promising catalytic performance of nanocatalysts. Therefore, some strategies like combination of magnetic nanocatalysts with different sources of H_2_O_2_ production should be developed. In the meantime, the approving biodegradability and biocompatibility of magnetic nanocatalysts should be considered to guarantee their potential safety *in vivo*.

Magnetic NPs with different physicochemical properties synthesized in a number of routes can be used as effective catalysts for organic reactions to develop heterocyclic compounds as potential anticancer drugs. Furthermore, their magnetic characteristics enable the simple and effective separation of the nanocatalyst using a magnetic MF to be reutilized up to several times without any remarkable changes in the initial catalytic function. These advantages can be carried out both in aqueous and non– aqueous environments. Additionally, the activity of fabricated compounds can be tested against a wide range of cancers. Furthermore, magnetic NPs can be integrated in the form of nanocomposite to develop the superparamagnetic properties and magnetization even at ambient temperature. The fabricated magnetic nanocomposite could demonstrate potential catalytic activity as a novel heterogeneous magnetic agent for the development of some heterocyclic compounds with profound anticancer activities.

Additionally, there are a number of approaches to selectively activate anticancer agents in the tumor site by means of nanocatalyst to reduce the relevant adverse effects. For instance, in the case of magnetic nanocatalysts, external MFs are utilized to target the magnetic nanocatalyst in the selected tissue, which can then catalytically result in activation of Fenton reactions.

**Fig. 5:**
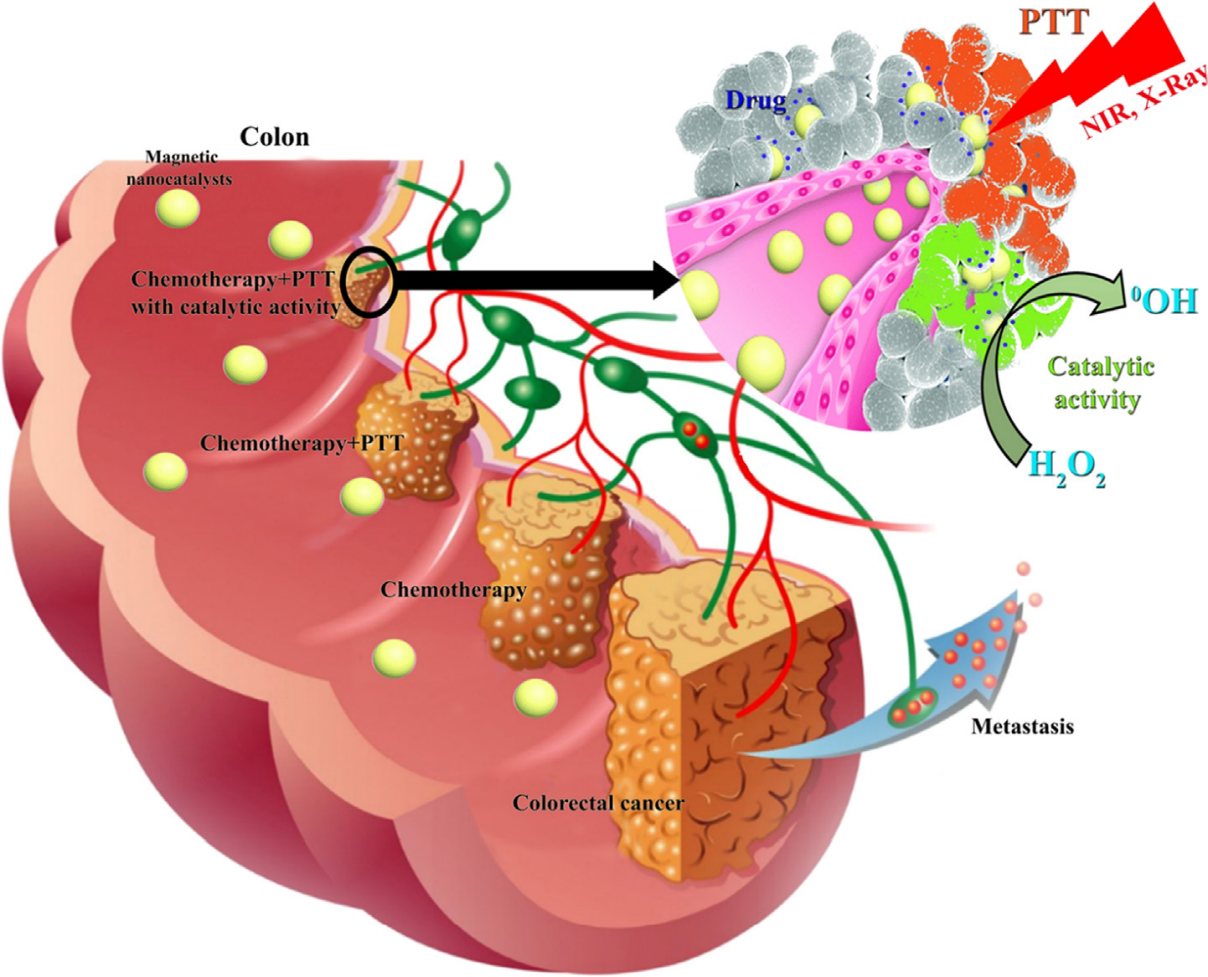
PTT in combination with magnetic nanocatalyst could be used as a potential platform in augmentation of Fenton chemical reaction for tumor therapy.

Also based on the specific properties of TME, a new inception of PTT in combination with magnetic nanocatalyst could be a promising approach in augmentation of Fenton chemical reaction for nanocatalytic tumor therapy (Fig. 5). These systems change the performance of nanocatalytic Fenton reaction for production of OH^•^ and enhancing the tumor mortality at a subsequent time. Indeed, GOD for generation of large amounts of H_2_O_2_ and Fe_3_O_4_ ‐modified with photothermal inducer‐based nanocatalyst can be fabricated to attain diagnostic imaging‐guided and photothermal‐improved nanocatalytic tumor therapy. Interestingly, the high photothermal‐turning efficacy of the inducer increases the defined tumor temperature to dramatically speed up and ameliorate the nanocatalytic activity, which potentially results in outstanding synergistic anticancer activities with minimal adverse impacts.

## Conclusion

The wide application of magnetic nanocatalysts derives from the fact that these agents have provided very encouraging outcomes in the synthesis of heterocyclic compounds as potential anticancer drugs as well as selective cancer treatments in several preclinical studies. In fact, magnetic nanocatalysts either alone or in conjugation with other NPs/molecules can be used as multiple promising nanocatalysts in synthesis of heterocyclic compounds based on magnetic NPs-catalyzed reactions, targeted drug delivery and facilitated Fenton reaction for cancer therapy. Nevertheless, only limited attempts have been done to translate the preclinical achievements of these smart agents to the clinics. Indeed, several challenges such as rapid metabolism, limited bioavailability and biodegradability should be taken into account in advance in order to bring magnetic nanocatalysts from the bench to the bedside. Pharmaceutical companies should try to overcome these drawbacks by reformulating nanocatalysts such as conjugation with other NPs or natural compounds.

## Key Scientific Concepts of review

This review may pave the way for well-organized translation of magnetic nanocatalysts in cancer therapy from the bench to the bedside.

## Declaration of Competing Interest

The authors have none to declare.
